# Dissemination of information to General Practitioners: a questionnaire survey

**DOI:** 10.1186/1471-2296-5-27

**Published:** 2004-11-30

**Authors:** Padma Moorjani, Heather Fortnum

**Affiliations:** 1 MRC Hearing and Communication Group, University of Manchester, Manchester, UK; 2 Trent Research and Development Support Unit, University of Nottingham, Nottingham, UK

## Abstract

**Background:**

Early identification of permanent hearing impairment in children enables appropriate intervention which reduces adverse developmental outcomes. The UK Government has introduced a universal hearing screening programme for neonates. All involved health professionals, including those in Primary Care, need to be aware of the service to enable them to offer appropriate support to their patients. A programme of information dissemination within Primary Care was therefore undertaken. The aim of the current study was to determine the extent to which the information had reached General Practitioners (GPs), the GPs' preferred mode of dissemination and the sources from which GPs accessed information

**Methods:**

Postal questionnaire survey of a randomised sample of 1000 GPs in the Phase I pilot sites of the Neonatal Hearing Screening Programme (NHSP).

**Results:**

Responses were received from 54.2% of the sample. Just under 50% of those responding had received information, 62.2% of respondents said they would like to receive more information and the preferred methods of dissemination were the written word and web-sites to allow access when needed. Few GPs perceive themselves to have a core role in the delivery of the NHSP and thence a need for knowledge in the subject. Many are keen to delegate detail to a third party, usually the health visitor, who has traditionally had responsibility for hearing screening.

**Conclusions:**

Dissemination efforts for service developments of relevance to GPs should concentrate on advertising a website address via brief but memorable posted literature and/or articles in relevant journals and magazines. The website should be GP-friendly, and have a dedicated area for GPs including information of specific relevance and downloadable information sheets.

## Background

Approximately 1.65 per 1000 babies born in the UK have a significant permanent hearing impairment (>40 dB HL) detectable at birth [1]. Early recognition and appropriate intervention can reduce the proven negative impact on the development of communication skills [2]. Thus, based on robust evidence [3], in June 2000 the UK Government announced an initiative to introduce effective and appropriate screening for women and children by 2004 [4] which included the implementation of a novel screen for hearing impairment for all newborns in England. Although targeted hearing screening has been in place in some areas for several years, the introduction of a universal service at birth and the proposal to abandon the health visitor distraction screen at the age of 8 months, has implications for the knowledge General Practitioners (GPs) are now expected to have. Thus a crucial part of the implementation of the new service was to provide information to GPs and other primary care staff. Previous studies addressing the issue of the most appropriate method of dissemination of information to GPs are inconclusive [5]. Rapidly developing technology is adding further methods [6], the ease of which may adversely contribute to the overload.

This study uses the introduction of the Newborn Hearing Screening Programme (NHSP) to illustrate the issues around dissemination to GPs [6,7]. Introduction of a novel screening service is a good example because a screen is only the beginning of a long process that integrates testing, diagnosis, intervention and monitoring [8], and in the case of permanent hearing impairment, long-term follow up. The National Screening Committee recognises information dissemination to be key to effective delivery of screening programmes stating that, "There is a need for all those involved in service delivery to understand the objectives and principles of the relevant screening programme(s) and be able to adequately interpret and explain the results and implications of the screening tests to patients as appropriate." [4] Primary care professionals are often the most accessible people with whom patients, in this case parents, can raise their questions.

The phased implementation of NHSP in the UK began in 2001 in 23 pilot sites and was accompanied by a campaign of information dissemination including distribution of leaflets and posters to all general practices, the development of a dedicated website and appointment of local co-ordinators. The aim of the current study was to determine the extent to which the dissemination information had reached the GPs, the GPs' preferred mode of dissemination and the sources from which GPs accessed information.

## Methods

We designed and piloted a one-page questionnaire [see Additional file 1] and used it to conduct a postal questionnaire survey early in 2003 of 1000 general practitioners in ten purposively sampled primary care trusts implementing the NHSP in the pilot sites. Five of the sites were those implementing the screen as a community based service and the remaining five were implementing the screen as a hospital based service sampled to match the community sites for annual birth rate. In each site 100 GPs were randomly selected to receive the questionnaire incorporating the restriction of only one questionnaire per practice. Identification of non-respondents was not possible as all information on returned questionnaires was anonymous. A reminder would have involved a total mailing. This option was rejected for reasons of inappropriate mailings to those GPs who had responded and cost.

## Results

### Response rate

Of the 960 questionnaires not returned by the Post Office and therefore assumed to have reached their destination, 520 (54.2%) were returned at least partially completed. The answers from the questionnaire are summarised in Table 1.

**Table 1:** Positive response to questionnaire items

	Total number of responses	Positive response	
		N	%
**Information received:**			
**Received any information**	506	252	49.8
Seen leaflets	508	125	24.6
Visited website	505	12	2.4
Got poster	495	64	12.9
Knows contact details of local co-ordinator	503	78	15.5
**Information requested:**	429	267	62.2
***Via written information***		*104*	*39.0*
***Via website***		*82*	*30.7*
***Via seminars***		*39*	*14.6*
***Via open day***		*16*	*6.0*
***Via meetings***		*15*	*5.6*
***Via e-mail***		*4*	*1.5*
Information sources:			
**Meetings:**			
**Existence of local GP forum**	436	268	61.5
***Attend local GP forum***		*39*	*14.6*
**Attend local child health seminar**	437	84	19.2
**Journals read:**			
**British Medical Journal**	457	363	79.4
**British Journal of General Practice**	457	142	31.1
**The Lancet**	457	4	0.9
**Update**	457	194	42.5
**BMA News**	457	165	36.1
**PCT Newsletter**	457	144	31.5
**Practitioner**	457	130	28.4
**Pulse**	457	32	7.0
**GP**	457	13	2.8
**Doctor**	457	6	1.3

### Information received

The responses indicate that just less than half of our sample had received any information about NHSP. Of those, half had seen (but not necessarily read) the leaflets but only 2.4% of responders (4.8% of those receiving information) had visited the website.

### Information requested

When asked if they would like more information about NHSP 62.2% said yes. Of those, the route most commonly cited was via the written word (39.0%) presumably leaflets and letters but 82 (30.7%) requested information via a website. Meetings and e-mail notification were not popular preferences.

### Information sources

To ensure that information reaches the target audience we should consider from which sources they get their information. Here it was clear that attending meetings is not common, less than 20% of respondents attended the local GP forum or child health seminar but GPs do read journals and magazines, notably the British Medical Journal (79.4%), Update (42.5%) and the British Journal of General Practice (31.1%).

### Open comments

In open comments (N = 84 respondents, 16.6%) it appeared that many of those that had received information had actually heard about the programme from the media or their own patients. A key theme in the comments was the delegation of responsibility for the knowledge to a colleague. While few GPs perceive themselves to have a core role in the delivery of the NHSP and thence a need for knowledge in the subject, many are keen to delegate detail to a colleague, usually the health visitor, who has traditionally had responsibility for hearing screening.

## Discussion

The response rate of more than 50% is acceptable for a survey of GPs. A second mailing might have improved this but its advantage was weighed against the attraction of anonymity. To minimise the number of questions and encourage response, no demographic details were requested. Analysis of any response bias by age of practitioner, size of practice etc was therefore not possible. A response might be more likely from people who knew something of the subject and our estimates for the level of awareness might therefore be high but it is likely that the views expressed concerning resources accessed and preferred channels of dissemination are representative.

Although considerable effort had been expended in distributing letters, leaflets and posters to every general practice, less than half the respondents said they had seen it. It is possible that mail had been screened and intercepted by administrative staff in the practice or rejected as "junk mail" by the GP. It is important therefore to ensure such mailed information is identified as relevant. Some health service developments will not be high on the average GP's agenda. In terms of neonatal hearing screening s/he will see only 20 newborns per year on average and approximately one permanently hearing-impaired child in a working lifetime.

Nearly two-thirds of respondents said they would like to receive more information even though many complained about "information overload". This is a weakness inherent in surveys of this nature where stated attitudes may reflect socially desirable responses rather than respondents' true attitudes. Further exploration of this issue using qualitative techniques of personal interview would contribute to the general issue of information dissemination to GPs.

GPs cannot know everything about everything and do not necessarily need to but they do need to know where they can find the relevant information and to be able to access it easily [9]. Our survey indicates that brief written information combined with the internet would be useful routes to use.

## Conclusions

We suggest that any dissemination effort for service developments of relevance to GPs should concentrate on advertising the website address via brief but memorable posted literature and/or articles in relevant journals and magazines. It is also crucial that any website be GP-friendly by having a dedicated area for GPs including information of specific relevance and downloadable information sheets.

## Competing interests

The author(s) declare that they have no competing interests.

## Authors' contributions

PM designed and carried out the survey, performed the analyses and wrote the original report. HF supervised the design and implementation of the study and drafted the manuscript. Both authors read and approved the final manuscript.

## Pre-publication history

The pre-publication history for this paper can be accessed here:



## Supplementary Material

Additional File 1 A survey of awareness of the NHS Newborn Hearing Screening Programme (NHSP) amongst General Practitioners (GPs) Questionnaire Click here for file
