# Supplementary material for: Dissemination of information to General Practitioners: a questionnaire survey
Source: BMC Fam Pract. 2004 Nov 30;5:27. doi: 10.1186/1471-2296-5-27 (PMC539264; doi:10.1186/1471-2296-5-27)
Supplement: Additional File 1 — A survey of awareness of the NHS Newborn Hearing Screening Programme (NHSP) amongst General Practitioners (GPs) Questionnaire [file 1471-2296-5-27-S1.doc]

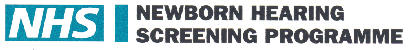


1. **Have you received any information about the Newborn Hearing Screening Programme (NHSP) ?**

**YES [ ] NO [ ]**

1. **Have you seen any of the following:**

## (a) NHSP information leaflets? YES [ ] NO [ ]

## (b) Have you previously visited the NHSP website (www.nhsp.info)? YES [ ] NO [ ]

## (c) NHSP poster YES [ ] NO [ ]

## if yes… Have you got it displayed in your practice? YES [ ] NO [ ]

1. **Do you have contact details of your local NHSP co-ordinator? YES [ ] NO [ ]**
2. **Have you been asked about NHSP by any of your patients? YES [ ] NO [ ]**
3. **At what age does the first hearing screen take place.**

(a) < 2weeks [ ] (b) 2-6weeks [ ] (c) 6-12weeks [ ] (d)>12weeks [ ]

(e) other [ ]

**6. Does your practice have meetings with the following ? (Please tick appropriate box)**

## (a) Health Visitors [ ] (b) District Nurse [ ] (c) Community Midwives [ ]

**7. If you have a local GP forum…**

How often does it meet? (a) weekly [ ] (b) monthly [ ] (c) Other…………………..

Do you attend this meeting? **YES [ ] NO [ ]**

###### How often do you attend this meeting? ………………………………

**8. Do you attend the Child Health Seminar in your area? YES [ ] NO [ ]**

1. **Which of the following do you read on a regular basis:(Please tick appropriate box)**

#### The British Journal of General Practice (BJGP) [ ] The Practitioner [ ] Update [ ]

#### BMA news [ ] BMJ [ ] Other (please specify) …………………………

**10. Would you like to receive more information about NHSP YES [ ] NO [ ]**

**11. How could this be done?**

(a) Provide more web base information [ ] (b) Seminars [ ] (c) Open day [ ]

(d) Other (please specify) ……………………..

**12. Do you feel you need more training about NHSP YES [ ] NO [ ]
if yes,** please specify ……………………………

**Thank-you for your co-operation. Please feel free to make any further comments overleaf.**

# **A survey of awareness of the NHS Newborn Hearing Screening Programme (NHSP) amongst General Practitioners (GPs)**

Please complete the questions below, and return in the envelope provided to;
Padma Moorjani, MRC Institute of Hearing Research, University Park, Nottingham NG7 2RD

**Name of Practice …………………………** **Postcode …………………………**

Practice list size ………………………… Number of partners ……………

**Website:** [www.nhsp.info](http://www.unhs.org.uk/)


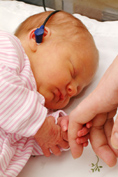


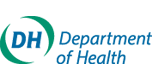


**Comments.**
